# Overcoming Bandwidth Limitations in Wireless Sensor Networks by Exploitation of Cyclic Signal Patterns: An Event-triggered Learning Approach

**DOI:** 10.3390/s20010260

**Published:** 2020-01-02

**Authors:** Jonas Beuchert, Friedrich Solowjow, Sebastian Trimpe, Thomas Seel

**Affiliations:** 1 Department of Engineering Science, University of Oxford, Oxford OX1 2JD, UK; 2 Intelligent Control Systems Group, Max Planck Institute for Intelligent Systems, 70569 Stuttgart, Germany; fsolowjow@is.mpg.de (F.S.); trimpe@is.mpg.de (S.T.); 3 Control Systems Group, Technische Universität Berlin, 10587 Berlin, Germany; seel@control.tu-berlin.de

**Keywords:** event-triggered state estimation, Gaussian processes, communication networks, bandwidth limitations, motion tracking, inertial measurement units, body area networks, physiological signals, data transmission protocols

## Abstract

Wireless sensor networks are used in a wide range of applications, many of which require real-time transmission of the measurements. Bandwidth limitations result in limitations on the sampling frequency and number of sensors. This problem can be addressed by reducing the communication load via data compression and event-based communication approaches. The present paper focuses on the class of applications in which the signals exhibit unknown and potentially time-varying cyclic patterns. We review recently proposed event-triggered learning (ETL) methods that identify and exploit these cyclic patterns, we show how these methods can be applied to the nonlinear multivariable dynamics of three-dimensional orientation data, and we propose a novel approach that uses Gaussian process models. In contrast to other approaches, all three ETL methods work in real time and assure a small upper bound on the reconstruction error. The proposed methods are compared to several conventional approaches in experimental data from human subjects walking with a wearable inertial sensor network. They are found to reduce the communication load by 60–70%, which implies that two to three times more sensor nodes could be used at the same bandwidth.

## 1. Introduction

**Figure 1:**
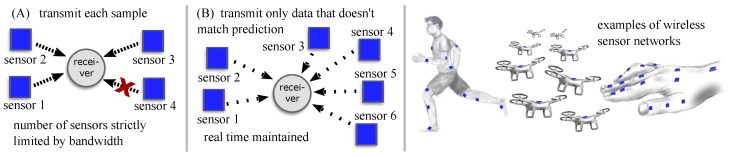
In wireless sensor networks, intelligent real-time communication protocols can reduce the communication load and enable the use of more sensors.

Real-time data transmission is important for a large range of applications. If multiple signals must be transmitted by a number of agents in a wireless network, bandwidth limitations impose restrictions on the number of agents that can transmit their signals in real time, as illustrated by Figure 1. One example is estimation and transmission of motion states in a wearable inertial sensor network. These systems are commonly used to provide biofeedback [1,2] or to control robotic systems [3,4] and neuroprostheses [4,5]. In such a network, a wireless inertial sensor is attached to each body segment of interest and sends measurements of its orientation to a receiving node. This receiver is typically a central unit that combines the measurements of multiple body segments to determine joint angles and similar motion parameters [6].

Whenever quick motions of several body segments are tracked, the available bandwidth of the wireless network imposes limitations that lead to a trade-off between the number of sensors and the rate at which they can send their data in real time. In the worst case, this leads to cables being used despite many disadvantages [7].

One main reason for these bandwidth limitations is that standard protocols transmit each measured sample of each signal even if it can be estimated accurately from previously transmitted samples. Intelligent protocols aim at exploiting such signal properties to reduce the communication load and thereby enable the use of more sensors or higher transmission rates, cf. Figure 1. Since the energy consumption of wireless communication is significant [8,9], a reduced communication load can also help to decrease battery sizes or increase use time.

Communication load can be reduced in two different ways:

(i) The number of transmitted values (bits) per sampling instant can be reduced.
(ii) The number of sampling instants with communication can be reduced.

While the first approach only reduces the payload of a data packet, the second approach aims at saving an entire data packet. Our main focus is, therefore, on the second approach. However, we will compare both approaches later on.

For obvious reasons, reducing the communication load should not be achieved at the cost of jeopardizing the accuracy of the transferred signal. Hence it is desirable to assure that the signal that is estimated or reconstructed in real time on the receiver side differs from the original signal only to a small, well-defined extent. To propose and validate methods that minimize the communication load while assuring such a small error bound is the primary goal of this article.

When a signal is approximately constant or linear in time, it seems straight-forward to accurately estimate the current sample from previously measured ones. However, if the signal varies in a less easily predictable manner, it is more difficult to find signal properties that can be exploited. In the present contribution, we consider signals that are approximately locally periodic. Approximately means that the signal can be well approximated by repetitions of a periodic pattern. Locally means that this pattern might change slowly or even suddenly, and for some time periods there might be no periodic pattern at all. Many physiological signals exhibit this approximate local periodicity, for example respiratory, cardiopulmonary, and EMG data [10], as well as inertial measurement data from periodic motions such as walking, cycling, and swimming. Even though the shape an ECG curve or the motion of the leg during swimming might change slowly or even suddenly, a very large portion of the signals is typically well described by cyclic patterns, and completely irregular episodes are rare.

We will aim at exploiting this approximate local periodicity by identifying the patterns in real time and transmitting only the data samples that cannot be adequately estimated from that pattern and previously transmitted data. To this end, we will use and extend recently proposed event-triggered learning (ETL) methods for cyclically excited systems [11], and we will validate the performance of the proposed methods with respect to more conventional approaches. The novel contributions of the present article are:

- We extend ETL to multidimensional nonlinear system with cyclic excitation and a non-Euclidean state space. The states of the system are unit quaternions that represent body segment orientations.
- We propose an ETL algorithm that uses Gaussian process regression (GPR) for prediction and thereby reduces the communication load that is associated with model updates.
- We validate the proposed methods using real measurement data and compare the performance to those of more conventional or fundamentally different approaches, such as compression by adaptive differential pulse code modulation.
- We apply the method to data from a sensor network and investigate the effect of network delays and a limitation of the number of transmission channels.

To the best of our knowledge, none of these contributions has been considered or presented in previous research.

An overview of the state of research is provided in Section 1.1. In Section 2, we describe the specific application and the addressed problem. New methods that address the given problem are proposed in Section 3. The data-based validation is described and the results are discussed in Section 4 and Section 5, respectively. Finally, Section 6 provides conclusions.

### 1.1. Related Work

Several methods have been proposed to reduce the communication load in wireless networks. For sake of simplicity, consider only the communication between one sender and one receiver, which might be part of a larger network. The sender measures the state of a system periodically, and the receiver must provide an estimate of that state in real time. All approaches that are considered below have in common that the sender applies some kind of compression algorithms before transmitting the measured data. Compression algorithms attempt to find structure in the measured data and remove redundant information. Subsequently, the receiver tries to reconstruct the original measured signal based on the transmitted data. We call the original signal values the *measurements* and the reconstructed signal values the *estimates*. Reconstruction might lead to an error between measurements and estimates, and this *reconstruction error* should be small.

The reviewed compression algorithms can be categorized based on two criteria: Firstly, algorithms that reduce the number of sampling instants with communication in contrast to algorithms that do not change this number, but reduce the amount of transmitted bits per sampling instant, and, secondly, lossless in contrast to lossy algorithms.

Event-based sampling (EBS [12,13,14,15]) and event-triggered state estimation (ETSE [9,16,17,18], sometimes referred to as model-based event-based sampling [19]) are well known lossy approaches to reduce the number of sampling instants with communication and guarantee a certain accuracy. Both can work in real time. In ETSE, the sender and the receiver independently predict the state measured by the sending agent. The prediction is based on an internal state and an invariant process model. The true measured state is only communicated and updates the internal states if a defined event indicates that the prediction is not accurate enough anymore. If the prediction is accurate, then the receiver uses the prediction as estimate.

The EBS algorithm is a special case of ETSE where the prediction is the last transmitted measurement. For example, ref. [12] applies EBS to a wireless wearable inertial measurement unit (IMU) network that communicates 3D accelerations and 3D angular velocities. The aim is to subsequently estimate joint angles. For a knee joint angle, they show that communication can be reduced by 66% compared to fixed-rate sampling, although the accuracy of the estimation is similar.

Furthermore, ref. [13] proposes EBS for transmission of orientations of human body segments in a wireless body sensor network. They use quaternions to represent these orientations and consider especially orientations of human feet. Depending on the parametrization, their method achieves a communication reduction by 70% at a root-mean-square-error (RMSE) of 7${}^{\circ }$ to 8${}^{\circ }$ or a RMSE of 1${}^{\circ }$ with a communication reduction by 5% to 10%. However, the error of the estimation is not guaranteed to be bounded.

The ETL algorithm [20,21] is an extension to ETSE. It can further reduce communication or increase accuracy if the system dynamics is time-variant. The sender has got the additional capability to learn a new model for prediction. Model learning is triggered and the estimated model is sent to the receiver if an event indicates that the system dynamics has changed.

Especially, ref. [11] proposes an ETL approach for cyclically excited systems. A setup with a single IMU on a human foot as sender, which provides a 1D pitch angle measurement, is considered. Using a base sampling rate of 50 Hz in an ideal network, communication can be reduced by more than 70% while the RMSE is below 1${}^{\circ }$ and the estimation error is bounded by 2${}^{\circ }$.

For a comprehensive study, ref. [22] uses IMUs to collect acceleration data during several running sessions. They compare the compression ratios, the additional transmission delays, and the RMSE of different lossless and lossy compression algorithms. Bzip2, zlib, and Lempel-Ziv-Welch (LZW) have got the advantage to be lossless compression algorithms, i.e., there is no reconstruction error. All three methods arrange consecutive data into packets before each packet is compressed individually. However, they reduce communication by 13% or less and introduce additional delays of at least 4 s. These delays prohibit real-time applications.

Lossy zlib and different (also lossy) wavelet compression schemes allow compression ratios of about 50% while introducing delays of more than 0.5 s. Again, these delays are too large for real-time applications.

Finally, ref. [23,24] propose modified adaptive differential pulse code modulation (ADPCM) for real-time compression. ADPCM does not change the number of sampling instants with communication, but reduces the amount of communication (bits) per sampling instant. For this, it codes difference values instead of absolute ones and adapts the quantization intervals to the signal. At every step, sender and receiver predict the measurement at the next sampling instant and only the difference between the prediction and the measurement is sent as update. This is beneficial because the range of the prediction error is expected to be much smaller than the range of the measurement value. Therefore, a smaller number of bits is required to achieve a similar accuracy. The prediction function and the quantization interval are adapted after each step. For validation, ref. [23,24] consider compression of raw signals of IMUs (acceleration, angular velocity, and magnetic field), but also the effect of compression on subsequent state estimation (attitude, heading, position, etc.). Using a sampling rate of 100 Hz, they reduce communication of the raw data by 60% while the RMSE of the subsequently estimated angles is 0.36${}^{\circ }$.

Further compression methods for sensor networks are proposed by [25,26] (Sensor LZW, S-LZW), ref. [27] (lossless entropy compression, LEC), ref. [28] (grouping and amplitude scaling), and [29] (time- and subject-adaptive dictionary). All these methods introduce delays of at least 1 s. In consequence, they are not suitable for real-time tracking.

To conclude, lossless algorithms are inefficient for motion data compression in real time. In the field of lossy compression algorithms, the number of methods that guarantee a certain accuracy and introduce no significant time delay is limited. The only approach that has already been studied for real-time compression of 3D orientation measurements does not guarantee a bounded error of the reconstructed signal and leads to errors of several degrees if a significant communication reduction should be achieved.

## 2. Specific Problem Setting

As explained above, we consider transmission of signals that exhibit unknown and time-varying cyclic patterns. In contrast to previous contributions, we consider multidimensional signals with nonlinear dynamics: three-dimensional orientations measured by a distributed network of inertial sensors.

### 2.1. Setup

**Figure 2:**
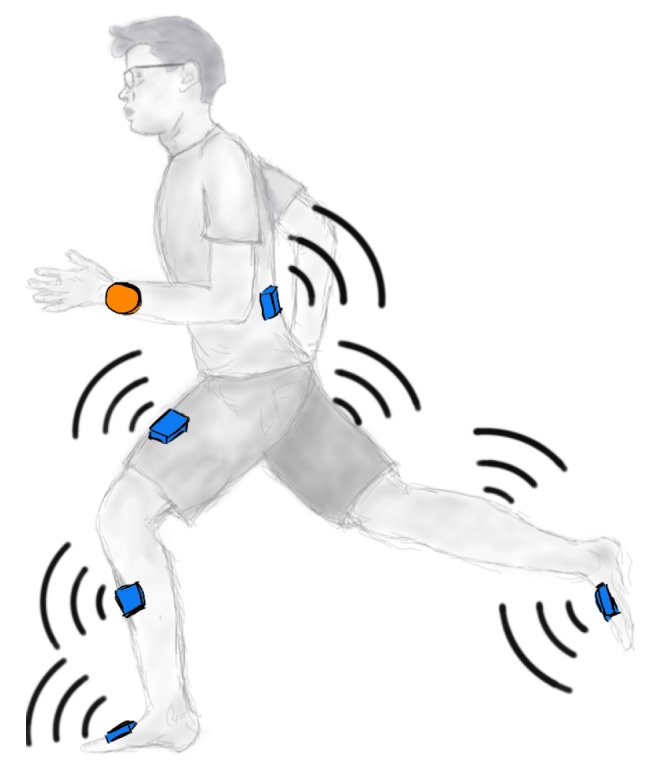
The human gait is assessed using a wearable wireless inertial sensor network consisting of seven sensors (blue) and one receiver (orange).

Consider a body sensor network consisting of one receiver and seven wearable sensor units, as illustrated in Figure 2. Each sensor unit comprises at least an IMU chip, a wireless communication module, and a microcontroller. The sensors are connected to the receiver via a star network; however, the following methods are not limited to this topology. The sensor network is used to track the motion of the lower limbs during gait. A fundamental assumption is that the motion is locally approximately cyclic as described in Section 1. This does not require constant steady-state gait; the subject might occasionally change the gait velocity and the walking style, for example by limping or tiptoeing.

Each sensor unit determines its orientation x[k]∈O at every sampling instant k∈Z. The sampling rate is 50 Hz. The set of all 3D orientations is denoted as O. In general, three real numbers are sufficient to describe all x∈O (cf. Section 3.1) and every number is coded with 16 bits. This leads to a payload of 48 bits for every data packet.

**Figure 3:**
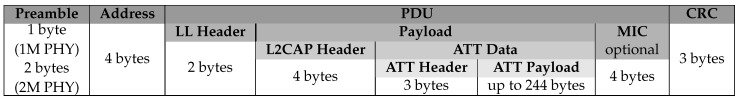
Packet format for uncoded Bluetooth 5 data [30].

Without loss of generality, we assume that Bluetooth 5 is employed for wireless data transmission, which is a widespread standard in today’s applications. We use the faster standard 2M PHY and do not enable encryption (MIC). These settings lead to a total overhead of 18 bytes = 144 bits per packet, cf. Figure 3.

### 2.2. Goals

In the given star network, and in several other network topologies, the network traffic is a superposition of many bilateral communications, in each of which one signal is being transferred from one sender to one receiver. We define goals for one such bilateral communication and demand that in a larger network these goals should hold for each bilateral communication.

The receiver must provide an accurate estimate $\hat{x}\left[k\right]\in O$ of the orientation at every sampling instant k=1,2, …,K in real time, where K∈N is the total number of samples in the measurement. A sufficiently intelligent communication protocol will achieve this goal without transmitting each measurement sample completely. We denote the number of transmitted data bits at each sampling instant with b[k]∈N and assess communication reduction by the following three measures:

- The number $P=\sum_{k=1}^{K}b\left[k\right]$ of transmitted data bits, i.e., the size of the ATT Payload (cf. Figure 3).
- The number $S=\sum_{k=1}^{K}{𝟙}_{\left\{\kappa :b\left[\kappa \right]>0\right\}}\left(k\right)$ of sampling instants with data transmission, where 𝟙:Z→{0,1} is the indicator function.
- The total number D=P+144S of transmitted bits including the Bluetooth 5 overhead.

On the one hand, all three numbers should be low. On the other hand, we want to achieve a small error between the measurement of the sender and the estimate of the receiver and introduce a fourth and a fifth measure for this error:

- The maximum absolute angle difference between the measured and the estimated body segment orientation $\max_{k}∢\left(x\left[k\right],\hat{x}\left[k\right]\right)$, i.e., the upper error bound.
- The RMS-value $\mathrm{RMSE}=\sqrt{\frac{1}{K}\sum_{k=1}^{K}{\left(∢\left(x\left[k\right],\hat{x}\left[k\right]\right)\right)}^{2}}$ of the error.

We define the angle difference $∢:O\times O\to \left[0,\pi \right]$ between two orientations as the shortest rotation angle that is necessary to rotate the first orientation onto the second one (cf. Section 3.1).

## 3. Methods

Before the introduction of three new methods to achieve the goals described in Section 2.2, we need an efficient way to represent 3D orientations of body segments and their rotations.

### 3.1. Orientation Representation

Orientations and rotations can be represented by the same mathematical structure since orientations can be seen as rotations with respect to a fixed reference frame. Such a structure is the set of unit quaternions [31,32]. Unit quaternions have got four components (a real part and three imaginary parts) that are real numbers and whose Euclidean norm is one. Specifically, we use augmented quaternions $q\in R^{4}$, i.e., quaternions in vector style. Their first component is the real part and the components two to four are the imaginary parts.

Quaternion multiplication is denoted by $⊗:R^{4}\times R^{4}\to R^{4}$. If $q_{1}\in R^{4}$ represents an orientation and $q_{2}\in R^{4}$ a rotation, then their product $q_{1}⊗q_{2}$ represents the orientation after the rotation. Furthermore, we define the angle difference $∢:R^{4}\times R^{4}\to \left[0,\pi \right]$ between two quaternions as the shortest rotation angle that rotates the first quaternion onto the second one. This is the arccos of the real part of the product of the first quaternion and the inverse of the second quaternion [33].

We consider orientations represented by unit quaternions as the states $x\left[k\right]\in R^{4}$ that are measured by the sender and must be estimated by the receiver at every sampling instant with index *k*. The orientation quaternions $x\left[k\right]$ are determined onboard from the IMU data using a sensor fusion algorithm, e.g., [34]. The estimated orientation quaternion of the receiver is denoted $\hat{x}\left[k\right]\in R^{4}$. To save 25% payload data, the sender always transmits only the imaginary part of the quaternion. Subsequently, the receiver restores the full unit quaternion using the Pythagorean theorem.

### 3.2. Event-Triggered Learning (ETL)

**Figure 4:**
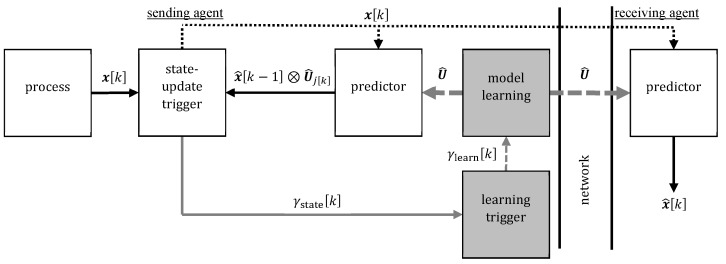
Block diagram of the event-triggered learning (ETL) architecture with one sender and one receiver [11,20]. The process provides the measured state $x\left[k\right]$ at every sampling instant *k*. The measured state is predicted based on the previous estimate $\hat{x}\left[k-1\right]$ and a trajectory model $\hat{U}$. If the prediction differs significantly from the measured state, then a state update is triggered ($\gamma_{\mathrm{state}}\left[k\right]=1$). If no state update is triggered ($\gamma_{\mathrm{state}}\left[k\right]=0$), then there is no network communication and the prediction is used as estimate $\hat{x}\left[k\right]$. Too frequent state updates indicate poor model quality and, therefore, trigger model learning ($\gamma_{\mathrm{learn}}\left[k\right]=1$). Then a new excitation trajectory $\hat{U}$ is learned by the sender and shared with the receiver.

The two related methods ETSE [12,13,14,15] and EBS [16,17,18,19] can be applied in the setup described in Section 2.1 to address the goals established in Section 2.2. Both are represented by the white blocks in Figure 4. The sender only sends samples $x\left[k\right]$ when a particular event occurs. However, the receiver provides the estimate $\hat{x}\left[k\right]$ at every sampling instant *k*, which requires it to perform a prediction whenever there is no communication. Specifically, the receiving agent and the sending agent independently predict the measurement $x\left[k\right]$ based on previous estimates and possibly a process model (ETSE). The predictions of both agents are identical. However, just the sender has got access to the true measurement $x\left[k\right]$. Therefore, it recognizes if the prediction is not good anymore, e.g., the difference between measurement and prediction is large. This is indicated by a binary state-update trigger $\gamma_{\mathrm{state}}\left[k\right]\in \left\{0,1\right\}$. If $\gamma_{\mathrm{state}}\left[k\right]=1$, then the sender communicates the true measurement $x\left[k\right]$, which updates the estimates $\hat{x}\left[k\right]$ of the sender and the receiver immediately.

The quality of the model is crucial for the accuracy of the predictions and, thus, for communication reduction, too. A drawback of ETSE is that it employs always the same model for prediction. This is not the case for ETL [20,21]; therefore, it can improve ETSE for systems with time-variant dynamics like in the considered setting with possibly changing motion patterns. Specifically, we use ETL for cyclically excited systems as describes in [11]. In comparison to ETSE, two new blocks are introduced at the sender’s side, which are shown in gray in Figure 4. A learning trigger detects if the dynamics of the measured process has changed and a model learning block uses previous measurements to identify a new model when the learning trigger fires. Subsequently, the sender updates the model of the prediction blocks of the sender and the receiver. On the one hand, sharing the model adds communication. On the other hand, if there is always a precise model, then the predictions are most precise and the number of state updates is smallest.

**Figure 5:**
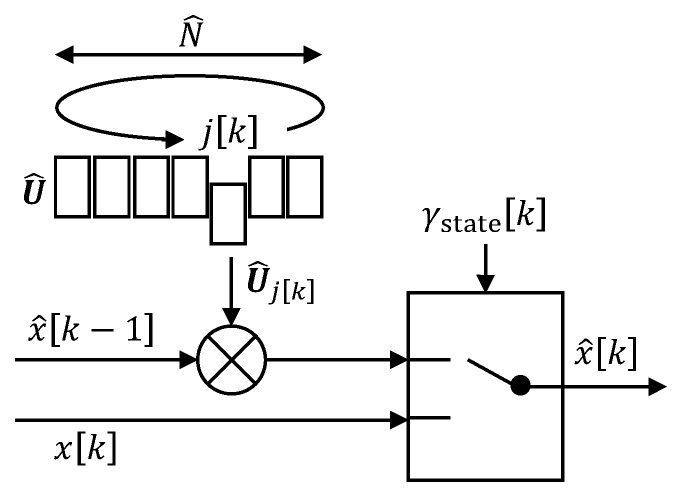
Flow chart of the trajectory-based event-triggered state estimation (ETSE). The model is a trajectory $\hat{U}$ of rotations (represented by quaternion increments) of one cycle with length $\hat{N}$. At every sampling instant, we multiply the previous orientation estimate $\hat{x}\left[k-1\right]$ with the rotation at index j[k] to obtain the prediction for sampling instant *k*. If the state-update trigger $\gamma_{\mathrm{state}}\left[k\right]$ is zero, then we employ this prediction as orientation estimate $\hat{x}\left[k\right]$; otherwise, we use the measurement x[k].

For the considered special case of repetitive quaternion signals as states, the model is a trajectory $\hat{U}\in R^{4\times \hat{N}}$ of rotations (quaternion increments) during one cycle with estimated cycle length $\hat{N}\in N$. The model of the rotation at the current step is the *j*-th column of $\hat{U}$ (cf. Figure 5). The index $j\left[k\right]\in N$ is increased after every step. If the end of the estimated model trajectory is reached ($j\left[k\right]=\hat{N}$) or learning is triggered ($\gamma_{\mathrm{learn}}\left[k\right]=1$), then $j\left[k\right]$ is reset to 1. Based on that, the event-triggered state estimation is

(1) $$\hat{x}\left[k\right]=\left\{\begin{array}{cc} \hat{x}\left[k-1\right]⊗{\hat{U}}_{j\left[k\right]} & \mathrm{if}\;\gamma_{\mathrm{state}}\left[k\right]=0\\ x\left[k\right] & \mathrm{if}\;\gamma_{\mathrm{state}}\left[k\right]=1 \end{array}\right..$$

We use the angle difference $d\left[k\right]=∢\left(x\left[k\right],\hat{x}\left[k-1\right]⊗{\hat{U}}_{j\left[k\right]}\right)$ between the measured quaternion and its prediction for the state-update trigger

(2) $$\gamma_{\mathrm{state}}\left[k\right]=\left\{\begin{array}{cc} 0 & \mathrm{if}\;d\left[k\right]<\delta \\ 1 & \mathrm{if}\;d\left[k\right]\geq \delta \end{array}\right.$$

with an error threshold $\delta \in R_{0}^{+}$.

Furthermore, we employ a binary learning trigger $\gamma_{\mathrm{learn}}\left[k\right]\in \left\{0,1\right\}$ to indicate when we want to update the model $\hat{U}$. Ideally, we trigger model updates if and only if the cyclic excitation has changed. Our trigger is based on the so-called inter-communication times, which are the times between two consecutive state updates. If the model no longer yields valid predictions of the current data, then the inter-communication times will decrease. We aim at detecting this decrease using the Kolmogorov-Smirnov test [35] as described in [11].

If model learning is triggered ($\gamma_{\mathrm{learn}}\left[k\right]=1$), then the excitation trajectory that was observed during the last cycle becomes the new model $\hat{U}$. As first step of the model learning, the cycle length $\hat{N}$ of the previously observed cycle is estimated. The estimation is done in the frequency domain (using the autocovariance of the measured states of a small number of previous cycles) and refined in the time domain with local optimization (Code and detailed description on http://www.control.tu-berlin.de/EventTriggeredLearning).

The rotations (quaternion increments) measured during the last cycle are denoted as $\Delta x\left[\ell \right]$, $\ell \in [k-\hat{N}+1,k]$. Then the new model trajectory is

(3) $$\hat{U}=\left[\begin{array}{ccc} \Delta x[k-\hat{N}+1] & \ldots & \Delta x[k] \end{array}\right].$$

To save more communication load, the trajectory $\hat{U}$ is compressed using polynomial regression applied individually to each of its imaginary parts. The sender transmits the compressed model to the receiver, which restores an approximation of the full trajectory.

### 3.3. Hierarchical ETL

**Figure 6:**
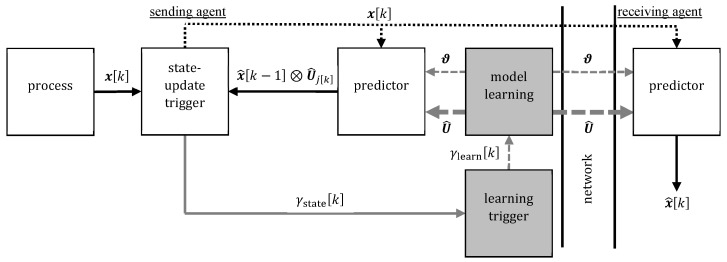
Block diagram of the hierarchical ETL architecture with one sender and one receiver [11]. In contrast to the standard ETL in Figure 4, triggered model learning can either lead to only an adjustment of certain parameters ϑ of the current model trajectory or to a completely new model trajectory $\hat{U}$.

Hierarchical ETL for cyclically excited systems [11] extends ETL (cf. Figure 6). In standard ETL, transferring the complete model $\hat{U}$ leads to a significant amount of communication whenever model learning is triggered. To address this disadvantage, we exploit the fact that small gait velocity changes can be well described by time-warping of the current quaternion trajectory, i.e., cycle length changes of the periodic excitation. If model learning is triggered, then the model learning block optimizes the two parameters cycle length $\vartheta_{1}=\hat{N}$ and phase shift $\vartheta_{2}$ of the current model $\hat{U}$ such that it fits the previously observed cycle best. Both parameters are estimated in the frequency domain (using the auto- or crosscovariance of the measured states of a small number of previous cycles) and corrected in the time domain with local optimization (Code and detailed description on http://www.control.tu-berlin.de/EventTriggeredLearning). This extended learning strategy allows for two hierarchical levels of model updates:

- Full model updates are updates of the whole trajectory $\hat{U}$.
- Small model updates adjust only a small number of parameters $\vartheta ={\left[\begin{array}{cc} \vartheta_{1} & \vartheta_{2} \end{array}\right]}^{T}$; in the examined problem setting, the current trajectory is warped to the new cycle length $\vartheta_{1}=\hat{N}$ and shifted by $\vartheta_{2}$ to obtain the new $\hat{U}$.

Full model updates should only be carried out if a small model update is not expected to improve the prediction performance sufficiently. For this, a binary learning-type trigger $\gamma_{\mathrm{full}}\left[k\right]\in \left\{0,1\right\}$ differentiates between a small and a full update [11]: The quaternion trajectory that would have been predicted with the warped and shifted model during the last cycle is calculated. Subsequently, this simulated trajectory is compared with the observed trajectory. The comparison is done by calculating the angle error between both trajectories at every point (cf. Section 3.1). The RMS-value $e\left[k\right]$ of the angle differences provides an estimate how good the model would fit after a small model update, i.e., just an update of the model parameters ϑ. If the error $e\left[k\right]$ exceeds a threshold $\alpha \in R_{0}^{+}$, then a full update is triggered

(4) $$\gamma_{\mathrm{full}}\left[k\right]=\left\{\begin{array}{cc} 0 & \mathrm{if}\;\gamma_{\mathrm{learn}}\left[k\right]=0\vee e\left[k\right]\leq \alpha \\ 1 & \mathrm{if}\;\gamma_{\mathrm{learn}}\left[k\right]=1\wedge e\left[k\right]>\alpha \end{array}\right..$$

Otherwise ($\gamma_{\mathrm{full}}\left[k\right]=0)$, the sender sends the parameters ϑ as small model update and both agents deform the previous trajectory to obtain the new model $\hat{U}$. If $\gamma_{\mathrm{full}}\left[k\right]=1$, then a full update of the model $\hat{U}$ is carried out as described in Section 3.2.

### 3.4. ETL with Gaussian Process Regression (GPR)

**Figure 7:**
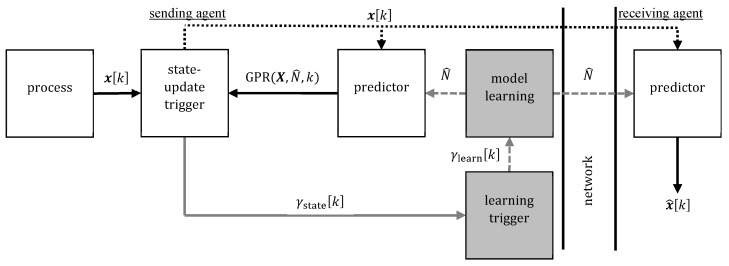
Block diagram of the ETL architecture with Gaussian Process Regression (GPR) for one sender and one receiver. In contrast to the standard ETL in Figure 4, predictions are GPR-based instead of trajectory-based. Consequently, the only information that is updated after model-learning is the estimated cycle length $\hat{N}$, which is the single variable hyperparameter of the GPR.

Hierarchical ETL reduces, but not eliminates, the drawback of standard ETL, which is that transferring the full model trajectory $\hat{U}$ requires the communication of a significant amount of values. An alternative method with much less model communication is introduced in this section. Its fundamental idea is to use a non-parametric machine learning method, which allows the receiving agent to learn the prediction model from the available measurement data instead of transferring a complete model. Specifically, Gaussian process regression (extrapolation) [36] implements the prediction block (cf. Figure 7) instead of the trajectory-based prediction (1).

Only state updates are available at the receiving agent and no additional states should be transferred. Therefore, the prediction at sampling instant *k* uses the previous p∈N measured and transferred states $X=\left[\begin{array}{ccc} x_{1} & \ldots & x_{p} \end{array}\right]$ with $x_{i}\in \left\{x\left[\ell \right]|\ell <k\wedge \gamma_{\mathrm{state}}\left[\ell \right]=1\right\}\forall i\in \left[1,p\right]$ and their indices $K=[\begin{array}{ccc} k_{1} & \ldots & k_{p} \end{array}]$ with $k_{i}\in \left\{\ell |\ell <k\wedge \gamma_{\mathrm{state}}\left[\ell \right]=1\right\}\forall i\in \left[1,p\right]$. In other words, $\left\{K,X\right\}$ is the training set for the GPR, which is adjusted whenever a state update occurs. The aim is to predict the output $\hat{x}\left[k\right]$ at index *k*. To make the problem suitable for GPR with one-dimensional outputs [36], we predict each imaginary part of $\hat{x}\left[k\right]$ independently of all others and make only sure that their norm is not larger than 1. Finally, the real part of the unit quaternion is obtained using the Pythagorean theorem (cf. Section 3.1).

A Gaussian process can be thought of as a distribution over a function space [36] and is uniquely determined by its mean function m:Z→R and covariance (kernel) function ϕ:Z×Z→R. Prediction (extrapolation) can be done with GPR if, in addition, the expected standard deviation $\sigma_{\mathrm{noise}}\in R_{0}^{+}$ of the noise of the outputs is defined. We set the mean function for each imaginary part to be piece-wise constant $m_{i}\left(k\right)=c_{i}\in R,i\in \left\{1,2,3\right\}$ as it is often done according to [36] because the kernel function provides enough flexibility to model significant deviations from the mean. The constant $c_{i}=\frac{1}{p}\sum_{\ell =1}^{p}X_{i+1,\ell }$ is updated after each state update since we assume that the empirical mean of the training data provides a good estimate of the true mean.

**Figure 8:**
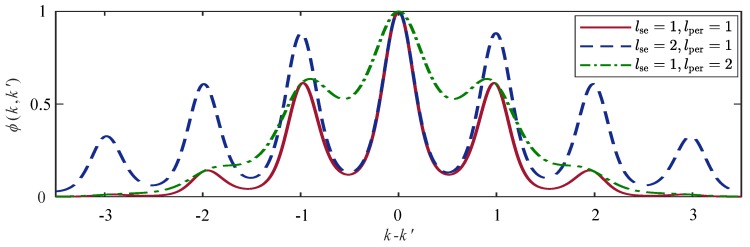
Locally periodic kernels (5) with different length scales $l_{\mathrm{se}}$ of the squared-exponential factor and length scales $l_{\mathrm{per}}$ of the periodic factor.

For the kernel function, three effects must be considered. On the one hand, data points whose indices are close to each other are expected to be correlated. On the other hand, every data point is assumed to be correlated with the data points one or more cycles before the current cycle. Additionally, it is reasonable to account for a gradual model change. All three points are adressed by using a locally periodic kernel

(5) $$\phi \left(k,k^{\prime }\right)=\sigma_{\phi }^{2}\exp\left(-\frac{2\sin^{2}\left(\frac{\pi \left|k-k^{\prime }\right|}{\hat{N}}\right)}{l_{\mathrm{per}}^{2}}-\frac{{\left|k-k^{\prime }\right|}^{2}}{2l_{\mathrm{se}}^{2}}\right),$$

which is the covariance between two state components at sampling instants *k* and $k^{\prime }$. The multiplication of a periodic kernel with the squared-exponential kernel leads to larger weights for data points in more recent cycles, compare [37,38] and especially [39] for an application involving human gait. The estimated cycle length $\hat{N}$ is the periodicity of the kernel. For good predictions, it must be known precisely. The lengthscale $l_{\mathrm{per}}\in R^{+}$ of the periodic kernel is fixed as well as the standard deviation (scaling factor) $\sigma_{\phi }\in R^{+}$. The lengthscale $l_{\mathrm{se}}\in R^{+}$ of the squared-exponential kernel depends on the cycle length $l_{\mathrm{se}}=\rho_{\mathrm{se}}\hat{N}$ with a fixed factor $\rho_{\mathrm{se}}\in R^{+}$. Figure 8 illustrates the influence of the individual parameters.

Just one model parameter is adapted online—the cycle length $\hat{N}$. Whenever model learning is triggered, the sender executes its estimation similar as described in Section 3.2 and Section 3.3, i.e., the cycle length is estimated in the frequency domain.

The estimated cycle length $\hat{N}$ is the only value which is transferred via the network during a model update. In consequence, each sender can send a maximum number of four values at a sampling instant (worst case of state update and model update together). This is much less than the amount of communication that is required by trajectory-based ETL to transmit a full model update of the trajectory $\hat{U}$ (cf. Section 3.2 and [11]) and, therefore, an advantage of GPR-based ETL because its communication load does not have salient peaks.

## 4. Experiments

We use the setup described in Section 2.1, i.e., we attach in total seven wireless IMUs (type XSENS MTWmathsizesmall) to a human body, two at the feet, two at the shins, two at the thighs, and one at the torso (cf. Figure 2), and record data sets during two different experiments:

- The first set (*variable*) contains approximately 4 min (180 steps) of simulated pathological gait with frequent style, speed, and ground inclination changes.
- The second set (*steady*) contains approximately 4 min (200 steps) of normal walking, at first with a speed of 2 km/h, then with 4 km/h.

During these experiments, each sensor unit records all measurement samples at a rate of 50 Hz, and a proprietary data recording software (MT Software Suite, XSENS) is used to transmit all measurement samples to a PC. Thereby, we obtain complete real-world application data sets on which we simulate and compare the proposed methods using MATLAB. For these simulations, we establish two fundamental assumptions: Firstly, the underlying network protocol accounts for channel noise and collision, re-sends samples if transmissions fail, and, thus, assures that no packets are lost. Secondly, we assume that processing and transmission delays are negligible. This is clearly an ideal environment. However, we examine the impact of channel number limitations and network delays in Section 4.5 and Section 4.6.

### 4.1. Parameterization

For all three ETL methods, the estimation error threshold for the state-update trigger (2) is $\delta =2^{\circ }$ (except for Section 4.2). This is, e.g., smaller than the error that is tolerated by neuroprosthesis controllers [5] and smaller than the state-of-the-art accuracy in foot orientation tracking [40]. Furthermore, the significance level of the Kolmogorov-Smirnov test as learning trigger is 5%, i.e., if the model is correct, then an (unwanted) model update is triggered with a probability smaller than 5%. Additionally, the test must fire for a minimum holding time of 0.35 s in order to make it more robust against false positives. Both parameters together lead to a good trade-off between the numbers of state and model updates in the considered application. For the compression of $\hat{U}$, polynomial regression with a degree of 18 is employed. In contrast to that, the full trajectory of a stride typically contains 40–80 samples. More parameters would increase the risk of overfitting.

For hierarchical ETL, the threshold of the learning-type trigger (4) is $\alpha =5^{\circ }$. This value balances the numbers of small and full model updates.

ETL with GPR-based predictions uses three fixed hyperparameters for the kernel, which are $\rho_{\mathrm{se}}=2$, $l_{\mathrm{per}}=0.5$, and $\sigma_{\phi }=20$, and obtained with cross-validation. For each prediction step, the GPR considers a dynamic training data set $\left\{K,X\right\}$ with a horizon of p=250. On the one hand, no data points with a significant covariance with respect to the current sample lie outside of this window; on the other hand, the computational effort is not too large. Finally, the noise’s standard deviation $\sigma_{\mathrm{noise}}$ is the same for each prediction. Because the value has got a physical meaning, it can be selected based on observations. With the available data, we establish an empirical mean of the standard deviation for different walking styles that is approximately $\sigma_{\mathrm{noise}}=0.01$.

### 4.2. Parameter Study

**Figure 9:**
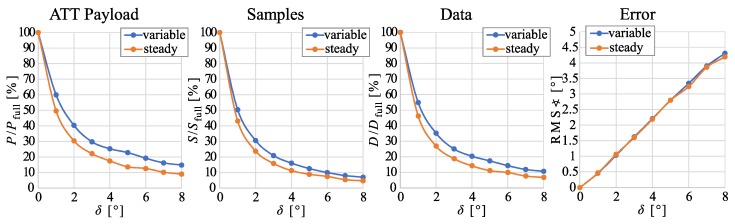
Performance impact comparison of different estimation error thresholds δ on hierarchical ETL. Data from feet-mounted sensors during 4 min of variable gait and steady walking is considered, respectively. The charts show the number *P* of transmitted data bits, i.e., the size of the ATT Payload, the number *S* of sampling instants with data transmission, and the total number *D* of transmitted bits including the Bluetooth 5 overhead with respect to full communication with the values $P_{\mathrm{full}}$, $S_{\mathrm{full}}$, and $D_{\mathrm{full}}$. In addition, the RMS-value of the angle difference between the measured and the estimated body segment orientation is displayed.

At first, we evaluate the influence of the angle difference threshold δ (the upper error bound) on the performance of hierarchical ETL (cf. Section 3.3). For simplification, we consider a setup with one foot-mounted sender and one receiver. Figure 9 shows the four performance measures introduced in Section 2.2 for nine different values of δ. Clearly, a higher communication reduction is achieved for steady gait then for variable gait. Additionally, the charts reveal that a slightly larger error threshold than $\delta =2^{\circ }$ enables even less data transmission for both types of gait.

### 4.3. Performance Comparison

We compare the performance of the three methods proposed in Section 3 (ETL, hierarchical ETL, and ETL with GPR) with four conventional methods from literature (full communication, decimation, IMA ADPCM, and EBS). This is done quantitatively based on simulations with real-world measurement data.

Firstly, full communication means that every sample is transmitted and no compression is applied.

Secondly, for decimation, we send only every *n*-th sampling instant with n∈N, i.e.,

(6) $$\hat{x}\left[k\right]=\left\{\begin{array}{cc} \hat{x}\left[k-1\right] & \mathrm{if}\;k\;\mathrm{mod}\;n>0\\ x\left[k\right] & \mathrm{if}\;k\;\mathrm{mod}\;n=0 \end{array}\right.,$$

where the expression mod:Z×N→N is the modulo operator. We choose the decimation factor n=2, i.e., every second sample is transmitted.

Thirdly, we examine IMA ADPCM (cf. Section 1.1) from [41,42]. The algorithm uses a fixed number of 4 bits per transmitted value. As described in Section 2.1, every measured quaternion component is coded with 16 bits. In consequence, the application of IMA ADPCM to all three imaginary parts reduces the payload by a factor of four.

Finally, EBS (cf. Section 1.1 and Section 3.2) is a special case of ETSE where the estimate $\hat{x}\left[k-1\right]$ at the last sampling instant is employed to predict the measurement $x\left[k\right]$, i.e.,

(7) $$\hat{x}\left[k\right]=\left\{\begin{array}{cc} \hat{x}\left[k-1\right] & \mathrm{if}\;\gamma_{\mathrm{state}}\left[k\right]=0\\ x\left[k\right] & \mathrm{if}\;\gamma_{\mathrm{state}}\left[k\right]=1 \end{array}\right..$$

Our EBS implementation uses the same state-update trigger (2) with the same estimation error threshold $\delta =2^{\circ }$ as ETL.

**Figure 10:**
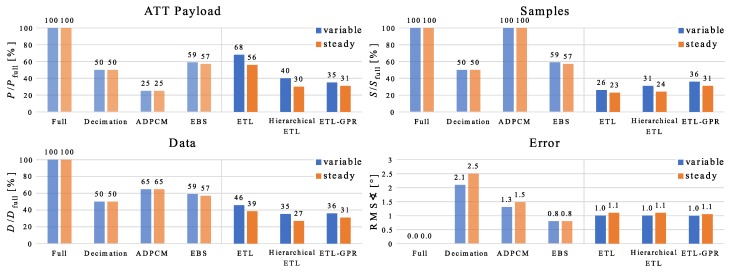
Performance comparison of seven methods. Data from feet-mounted sensors during 4 min of variable gait and normal walking is considered, respectively. The charts show the number *P* of transmitted data bits, i.e., the size of the ATT Payload, the number *S* of sampling instants with data transmission, and the total number *D* of transmitted bits including the Bluetooth 5 overhead with respect to full communication with the values $P_{\mathrm{full}}$, $S_{\mathrm{full}}$, and $D_{\mathrm{full}}$. In addition, the RMS-value of the angle difference between the measured and the estimated body segment orientation is displayed.

**Figure 11:**
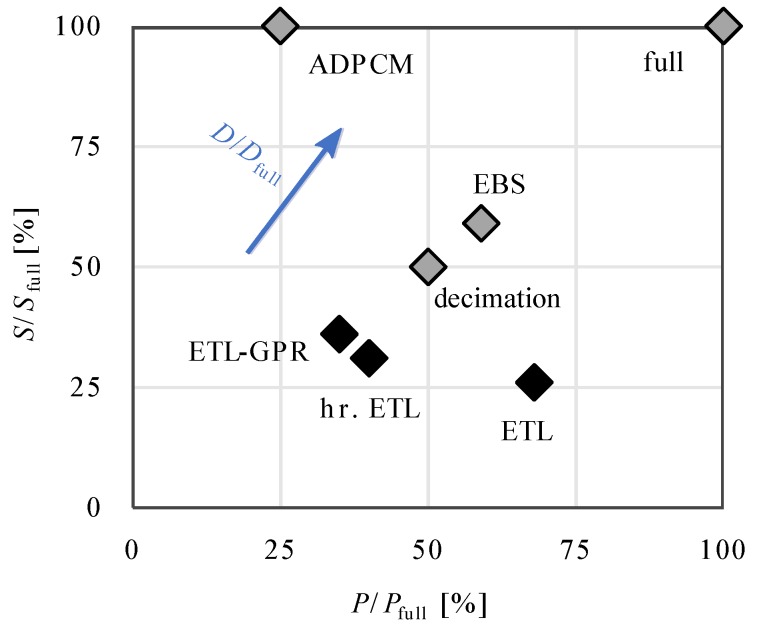
Number *S* of sampling instants with data transmission over the number *P* of transmitted data bits, i.e., the size of the ATT Payload, for seven different methods. The total number *D* of transmitted bits including the Bluetooth 5 overhead increases from the bottom left to the top right corner. Data from feet-mounted sensors during 4 min of variable gait is considered.

For simplification, we consider again a setup with one sender and one receiver and only feet (cf. Section 4.2). Later, Section 4.4 demonstrates that the results for sensors attached to other lower limb segments are qualitatively the same. Figure 10 shows the four performance measures introduced in Section 2.2 for all methods. The three novel approaches reduce the total amount of transmitted data more than the baseline methods for both, variable as well as steady gait; although, the resulting estimation errors are small with RMS-values of about 1${}^{\circ }$. Additionally, Figure 11 visualizes the trade-off between the payload and the number of transmitted packets for the different methods. While ADPCM reduces the number of sampling instants with communication most, ETL achieves the smallest payload. However, the two modified ETL methods (hierarchical ETL and GPR-based ETL) lead to the best compromises between both measures such that their total amount of transmitted data including the Bluetooth 5 overhead is the smallest.

### 4.4. Different Body Segments

**Figure 12:**
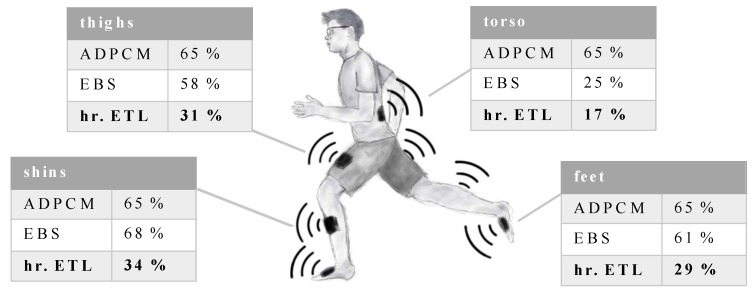
Total number *D* of transmitted bits including the Bluetooth 5 overhead with respect to full communication with $D_{\mathrm{full}}$ bits. Three of the methods—adaptive differential pulse code modulation (ADPCM), event-based sampling (EBS), and hierarchical ETL—are considered for four different types of body segments during 4 min of variable gait. The values for feet, shins, and thighs are averaged (left and right body segment).

In contrast to Section 4.2 and Section 4.3, we consider all seven IMUs as senders in a star network for the following analysis. Such a star topology with one receiver and multiple senders is common in biomedical applications and human motion tracking. Additionally, more complex network structures can often be decomposed into several star networks. For simplicity, we assume that the senders communicate at the same time with the receiver. Figure 12 shows the total amount of transmitted data as defined in Section 2.2 (including Bluetooth 5 overhead) with respect to full communication for the variable data set. The tables reveal that hierarchical ETL leads not only to a superior communication reduction in contrast to the baseline methods for feet, but also for other body segments.

### 4.5. Delayed Model Identification and Transmission

If ETL or hierarchical ETL with trajectory-based predictors is used, then transmission of full models causes significantly more communication than transmission of state updates. Additionally, model learning requires the most computational power of all steps of the proposed methods. Therefore, the aim is to examine how a potentially delayed identification and transmission of models affects the performance of the methods. For this, we assume ten sampling instants delay for model updates in ETL, i.e., a newly learned model is only available to the receiver ten sampling instants after learning was triggered. This is more than what is expected in practice due to communication and computation delays in modern communication networks. We carry out the same simulations as for Figure 12 to calculate $D/D_{\mathrm{full}}$. The result is that the percentage values for hierarchical ETL increase by 2% or less for all examined body segments. In consequence, they are still significantly smaller than the results for EBS and ADPCM.

### 4.6. Limited Number of Channels

Under poor conditions it can happen that not all senders can communicate with a single receiver at the same time. This fact has not been considered in the previous simulations and is, therefore, examined in the following. In reality, the number of IMUs that can communicate successfully at the same sampling instant is a random number. For simplicity, we define that this limit is constant. If an additional IMU attempts to communicate, then it must wait until the next sampling instant. In the simulations, the decisions which IMUs must wait are random.

**Figure 13:**
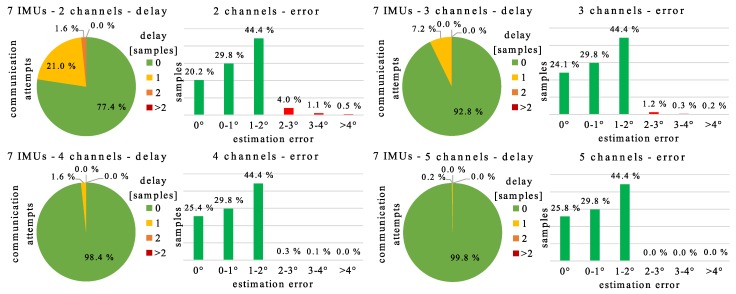
Simulation results for different assumed maximum channel numbers in a star network with seven inertial measurement units (IMUs) as senders. The pie charts show how often a sender can communicate immediately (delay 0) or must wait 1, 2, or more sampling instants until its communication attempt is successful. The bar charts show the distribution of the estimation error. We consider hierarchical ETL and 4 min of variable gait.

Figure 13 shows how often the case occurs that an IMU must wait for one or even more sampling instants when hierarchical ETL is simulated with the variable measurement data set (cf. Section 4) and different maximum channel numbers. If a state update cannot be transmitted immediately, then the error bound δ is violated. Figure 13 displays histograms of the magnitudes of these boundary violations in comparison to boundary compliances, too. If at least three channels can be used, then the error bound is never violated for more than one sampling instant by a single sender. If four channels or more are available, then the total amount of boundary violations is less than 0.5% and, in consequence, negligible.

## 5. Discussion

The experimental results are discussed in two stages. Firstly, we compare the three novel methods with the four baseline methods using the example of one foot-mounted sender and one receiver. Secondly, we analyze the properties of the best performing novel method in a larger sensor network.

### 5.1. Method Comparison

Decimation with factor two sends samples periodically and reduces the communication by 50%, but introduces a large estimation error. This is because the selection of the samples that the sender sends to the receiver is pre-determined. In contrast to that, EBS selects the transmitted samples online (event-based) to guarantee a bounded error with as little communication as possible. Therefore, this method is able to achieve a much smaller error with an almost comparable communication reduction. ETL reduces the amount of samples with communication even more without causing a much bigger RMSE. This is possible with model-based predictions and model updates, which account for time-variant behavior of the system dynamics. Furthermore, a hierarchical architecture improves ETL without compromising the error. This strategy exploits the fact that a velocity change can be described with only two parameters, which reduces the communication in case of model updates. Although the model trajectories after small updates might be less accurate than after a full update, the prediction error and, therefore, the number of state updates increases only a little. With the hierarchical extension, ETL with trajectory-based predictions shows a comparable performance as ETL with GPR with respect to communication reduction and the RMSE. However, a disadvantage of GPR is that it requires more computational resources. On the other hand, the communication load is more evenly distributed over the sampling instants. This is because the prediction with GPR is less precise than with trajectories, and more state updates occur, but model updates are much smaller.

In addition to the four quantitative performance measures, the existence of an upper bound on the estimation error is an important assessment criteria. Only EBS and the different versions of ETL guarantee such a bound while decimation and ADPCM can cause arbitrarily large errors.

Moreover, the quantitative measures for the performance of ADPCM differ significantly from the ones for the other methods. On the one hand, ADPCM reduces the payload per sampling instant most. On the other hand, it does not reduce number of sampling instants. Therefore, the overhead is not reduced and the total amount of communication does not shrink as much as with the ETL nethods.

### 5.2. Sensor Networks

We select hierarchical ETL for further evaluation in sensor networks with more than two agents because it is the novel method with the smallest amount of total communication in the previous contemplation. Firstly, hierarchical ETL shows a similar or superior performance for other body segments than feet; EBS, the baseline method with the best trade-off between communication reduction and estimation error, is not superior to hierarchical ETL for any segment.

Furthermore, we demonstrate that model update delays are no practical problem, i.e., the communication load does not increase significantly even for large delays. Additionally, the robustness against channel limitations is a special property of ETL, e.g., ADPCM cannot handle those because every sender must communicate at every sampling instant. With hierarchical ETL, we can use twice as many sensors as channels without overshooting the error bound for more than one sampling instant.

### 5.3. Limitations

The presented validation study has some limitations, which are briefly discussed in the following. Due to the small number of different experiments and subjects, the results provide only a qualitative proof of concept. They show the potential and elementary properties of the methods but do not yield precise performance quantification. For a detailed assessment, a more extensive study must be performed in a specific application scenario. The current approach of simulated-real-time processing of recorded data allowed us to investigate accuracy, communication load reduction and robustness to network delays independently and to gain insights into the performance of the algorithm in predefined situations. However, a detailed performance analysis in a specific application scenario will require testing of the proposed algorithms on embedded hardware with real-time wireless communication and uncontrolled bandwidth and delay variations. Performance should be evaluated also for a large number of agents in the network as well as for different communication protocols with different overheads and payloads.

Furthermore, it should be noted that the communication reduction achieved with hierarchical ETL in comparison to non-hierarchical ETL depends on the size of the large model update. In this work, its size is similar for all experiments. For a smaller full model update, the benefits of the hierarchical approach are expected to be limited.

### 5.4. Comparison with Related Work

In comparison to most of the existing algorithms for data compression in sensor networks (cf. Section 1.1), the proposed ETL methods work in real time. One of the methods that is covered in Section 1.1 and is also real-time-capable is ADPCM [23,24]. However, we demonstrated in Section 5.1 that ETL outperforms ADPCM on the given evaluation data.

Furthermore, ETL is preferable to the real-time-capable method presented in [13] (EBS), which achieves almost no compression at a RMSE of 1${}^{\circ }$ or leads to a five to ten times larger error for compression rates similar to those achieved by ETL.

Finally, the relative communication reduction of the proposed ETL methods is similar to the one achieved in [12] with EBS. However, the direct comparison of EBS and ETL on the same evaluation data in Section 5.1 revealed the performance increase that ETL achieves by exploiting the approximate periodicity of the signals. Moreover, the proposed quaternion-based approach leads to a general decrease of communication load in comparison to the approach in [12] because it transmits only three-dimensional signals (imaginary parts of unit quaternions) instead of six-dimensional measurements (3D accelerometer and gyroscope data, respectively). Both advantages come at the cost of increased computational effort.

## 6. Conclusions

The proposed methods reduce the communication load in sensor networks by exploiting the fact that physiological signals are often approximately cyclic. They account for the fact that these periodic patterns might change gradually or suddenly over time.

A major advantage is that ETL guarantees a user-defined upper bound for the estimation error. In the considered experimental data, the total communication load can be reduced by more than 60% or at least twice as many sensors can be used at transmission inaccuracies as small as one degree. The relative communication load reduction is expected to further improve for sampling rates at or above 100 Hz, which are commonly used in wireless IMU networks and desirable for analysis of fast and agile motions.

A disadvantage of ETL is that it requires more computational power than full communication. However, the power consumption due to communication can easily outweigh the power consumption spent on computation [8]. The total energy demand will be reduced in many application systems, and lighter batteries can be used or the time of use can be increased.

Future work will aim at large-scale validation of accuracy and reliability in selected application scenarios in wearable sensor networks and swarm robotics. In this context, dynamic network topology changes and multi-hop network topologies will be considered.

## References

[1] Giggins O.M., Persson U.M., Caulfield B. (2013). Biofeedback in rehabilitation. J. Neuroeng. Rehabil..

[2] Schwartz M. (2018). Biofeedback.

[3] Huen D., Liu J., Lo B. An integrated wearable robot for tremor suppression with context aware sensing. Proceedings of the IEEE 13th International Conference on Wearable and Implantable Body Sensor Networks (BSN).

[4] Schicketmueller A., Rose G., Hofmann M. (2019). Feasibility of a Sensor-Based Gait Event Detection Algorithm for Triggering Functional Electrical Stimulation during Robot-Assisted Gait Training. Sensors.

[5] Seel T., Werner C., Schauer T. (2016). The adaptive drop foot stimulator–Multivariable learning control of foot pitch and roll motion in paretic gait. Med Eng. Phys..

[6] Teufl W., Lorenz M., Miezal M., Taetz B., Fröhlich M., Bleser G. (2019). Towards inertial sensor based mobile gait analysis: Event-detection and spatio-temporal parameters. Sensors.

[7] Salchow-Hömmen C., Callies L., Laidig D., Valtin M., Schauer T., Seel T. (2019). A Tangible Solution for Hand Motion Tracking in Clinical Applications. Sensors.

[8] Lanthaler M. (2008). Self-Healing Wireless Sensor Network. https://www.cs.helsinki.fi/u/niklande/opetus/SemK07/paper/lanthaler.pdf.

[9] Suh Y. (2007). Send-on-delta sensor data transmission with a linear predictor. Sensors.

[10] Bronzino J.D., Peterson D.R. (2018). Biomedical Engineering Fundamentals.

[11] Beuchert J., Solowjow F., Raisch J., Trimpe S., Seel T. (2019). Hierarchical Event-triggered Learning for Cyclically Excited Systems with Application to Wireless Sensor Networks. IEEE Control Syst. Lett..

[12] Laidig D., Trimpe S., Seel T. (2016). Event-based sampling for reducing communication load in realtime human motion analysis by wireless inertial sensor networks. Curr. Dir. Biomed. Eng..

[13] Zhang T., Laidig D., Seel T. Stop Repeating Yourself: Exploitation of Repetitive Signal Patterns to Reduce Communication Load in Sensor Networks. Proceedings of the 18th European Control Conference (ECC).

[14] Miskowicz M. (2015). Event-Based Control and Signal Processing.

[15] Lemmon M. (2010). Event-triggered feedback in control, estimation, and optimization. Networked Control Systems.

[16] Shi D., Shi L., Chen T. (2016). Event-Based State Estimation.

[17] Trimpe S., D’Andrea R. (2014). Event-based state estimation with variance-based triggering. IEEE Trans. Autom. Control.

[18] Sijs J., Lazar M. (2012). Event based state estimation with time synchronous updates. IEEE Trans. Autom. Control.

[19] Liu Q., Wang Z., He X., Zhou D. (2014). A survey of event-based strategies on control and estimation. Syst. Sci. Control Eng. Open Access J..

[20] Solowjow F., Baumann D., Garcke J., Trimpe S. Event-triggered learning for resource-efficient networked control. Proceedings of the Annual American Control Conference (ACC).

[21] Solowjow F., Trimpe S. (2019). Event-triggered learning. arXiv.

[22] Cheng L., Hailes S., Cheng Z., Fan F.Y., Hang D., Yang Y. Compressing inertial motion data in wireless sensing systems—An initial experiment. Proceedings of the 5th International Summer School and Symposium on Medical Devices and Biosensors.

[23] Suh Y.S. (2009). Inertial and magnetic sensor data compression considering the estimation error. Sensors.

[24] Suh Y.S., Ro Y.S., Kang H.J. Inertial sensor data compression using modified ADPCM. Proceedings of the 11th International Conference on Control Automation Robotics & Vision.

[25] Arici T., Gedik B., Altunbasak Y., Liu L. PINCO: A pipelined in-network compression scheme for data collection in wireless sensor networks. Proceedings of the 12th International Conference on Computer Communications and Networks.

[26] Sadler C.M., Martonosi M. Data compression algorithms for energy-constrained devices in delay tolerant networks. Proceedings of the 4th International Conference on Embedded Networked Sensor Systems.

[27] Marcelloni F., Vecchio M. (2009). An efficient lossless compression algorithm for tiny nodes of monitoring wireless sensor networks. Comput. J..

[28] Gandhi S., Nath S., Suri S., Liu J. Gamps: Compressing multi sensor data by grouping and amplitude scaling. Proceedings of the ACM SIGMOD International Conference on Management of Data.

[29] Vadori V., Grisan E., Rossi M. Biomedical signal compression with time-and subject-adaptive dictionary for wearable devices. Proceedings of the IEEE 26th International Workshop on Machine Learning for Signal Processing (MLSP).

[30] Afaneh M. (2017). Bluetooth 5 speed: How to Achieve Maximum Throughput for Your BLE Application. https://www.novelbits.io/bluetooth-5-speed-maximum-throughput.

[31] Hamilton W.R. (1847). On quaternions; or on a new system of imaginaries in algebra. Lond. Edinb. Dublin Philos. Mag. J. Sci..

[32] Kuipers J.B. (1999). Quaternions and Rotation Sequences.

[33] Huynh D.Q. (2009). Metrics for 3D Rotations: Comparison and Analysis. J. Math. Imaging Vis..

[34] Seel T., Ruppin S. (2017). Eliminating the effect of magnetic disturbances on the inclination estimates of inertial sensors. IFAC-PapersOnLine.

[35] Massey F.J. (1951). The Kolmogorov-Smirnov test for goodness of fit. J. Am. Stat. Assoc..

[36] Rasmussen C., Williams C. (2006). Gaussian Processes for Machine Learning.

[37] Klenske E.D., Zeilinger M.N., Schölkopf B., Hennig P. (2016). Gaussian process-based predictive control for periodic error correction. IEEE Trans. Control Syst. Technol..

[38] Roberts S., Osborne M., Ebden M., Reece S., Gibson N., Aigrain S. (2013). Gaussian processes for time-series modelling. Philos. Trans. R. Soc. A Math. Phys. Eng. Sci..

[39] Dhir N. (2017). Bayesian Nonparametric Methods for Dynamics Identification and Segmentation for Powered Prosthesis Control. Ph.D. Thesis.

[40] Seel T., Graurock D., Schauer T. (2015). Realtime Assessment of Foot Orientation by Accelerometers and Gyroscopes. Curr. Dir. Biomed. Eng..

[41] IMA Digital Audio Focus and Technical Working Groups (1992). Recommended Practices for Enhancing Digital Audio Compatibility in Multimedia Systems. https://www.cs.columbia.edu/~hgs/audio/dvi/IMA_ADPCM.pdf.

[42] Rabiner L.R., Schafer R.W. (1978). Digital Processing of Speech Signals.

